# Luminescent Measurement Systems for the Investigation of a Scramjet Inlet-Isolator

**DOI:** 10.3390/s140406606

**Published:** 2014-04-09

**Authors:** Azam Che Idris, Mohd Rashdan Saad, Hossein Zare-Behtash, Konstantinos Kontis

**Affiliations:** 1 Faculty of Engineering, National Defence University of Malaysia, Kem Sungai Besi, Kuala Lumpur 57000, Malaysia; E-Mail: rashdan@upnm.edu.my; 2 School of Engineering, University of Glasgow, James Watt South Building, Glasgow G12 8QQ, Scotland; E-Mails: Hossein.Zare-Behtash@glasgow.ac.uk (H.Z.-B.); Kostas.Kontis@glasgow.ac.uk (K.K.)

**Keywords:** scramjet inlet, hypersonic, pressure-sensitive paint (PSP), stream-thrust

## Abstract

Scramjets have become a main focus of study for many researchers, due to their application as propulsive devices in hypersonic flight. This entails a detailed understanding of the fluid mechanics involved to be able to design and operate these engines with maximum efficiency even at their off-design conditions. It is the objective of the present cold-flow investigation to study and analyse experimentally the mechanics of the fluid structures encountered within a generic scramjet inlet at *M* = 5. Traditionally, researchers have to rely on stream-thrust analysis, which requires the complex setup of a mass flow meter, a force balance and a heat transducer in order to measure inlet-isolator performance. Alternatively, the pitot rake could be positioned at inlet-isolator exit plane, but this method is intrusive to the flow, and the number of pitot tubes is limited by the model size constraint. Thus, this urgent need for a better flow diagnostics method is addressed in this paper. Pressure-sensitive paint (PSP) has been applied to investigate the flow characteristics on the compression ramp, isolator surface and isolator sidewall. Numerous shock-shock interactions, corner and shoulder separation regions, as well as shock trains were captured by the luminescent system. The performance of the scramjet inlet-isolator has been shown to improve when operated in a modest angle of attack.

## 1. Introduction

**Figure 1.**
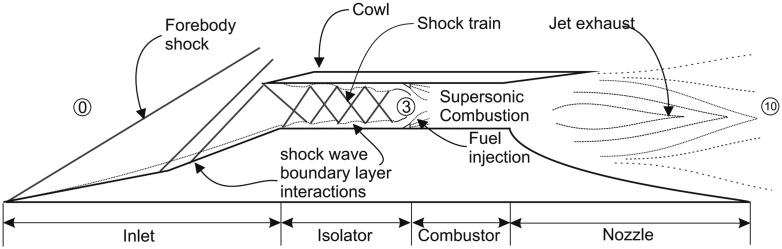
Generic schematic of a scramjet engine working principle where, typically, compression is provided between Station **0** and **3** prior to fuel injection and combustion.

The historical successful hypersonic flight of Boeing's X-51A in May 2013, has boosted the interest in a single- or two-stage-to-orbit spaceplane. The key enabler technology of this project is the supersonic combustion ramjet (scramjet) engine, which is capable of operating in the hypersonic speed regime. The scramjet engine borrows heavily from the ramjet working principle, where the need for a compression unit for the engine is provided by the strategically shaped inlet, eliminating the need for a compressor. In a ramjet engine, the inlet will compress the incoming supersonic flow into subsonic speeds suitable for combustion. However, at approximately Mach 5, it would be very inefficient if the flow is decelerated to subsonic; thus, combustion must be done supersonically [1]. A schematic of the generic scramjet working principle is provided in Figure 1.

Even though the scramjet concept has been in development since the late 1950s [2], much more work needs to be done to eliminate various hurdles and produce a robust prototype. Most of the hurdles are centred on the compression component, which is the inlet-isolator section. Some inlet flow phenomena that require urgent attention include: (1) boundary-layer transition; (2) shock wave-boundary layer interaction (SWBLI); (3) boundary layer separation; (4) shock-shock interactions; (5) glancing shock interactions; and many more.

Investigations of the various phenomena usually involve the schlieren technique for flow visualizations and pressure transducer measurement for meaningful quantitative analysis. For example, H*ä*berle and G*ü*lhan [3] analyzed the behaviour of internal shock structures inside an inlet-isolator using combinations of schlieren and static pressure measurements along the centreline of the model. Changes in the isolator shock structures can be detected with pitot rake pressure measurements [4]. The pitot rake positioned at the isolator exit (Station **3** in Figure 1) can provide the necessary flow properties to calculate the performance of an inlet. First demonstrated by Berstein and Haefeli [5,6] in the early 1950s, the average pitot pressure at the throat can be used in combinations with static wall pressure to deduce the inlet throat Mach number and stagnation pressure. The flow properties can then be used to calculate the inlet total pressure loss and kinetic energy efficiency. Extensive scramjet inlet experimental analyses done in the German Aerospace Center (DLR) utilised a similar pitot rake method for measuring inlet performance, albeit with some modifications on the equation [7–9]. The main problem of relying on pressure transducers for inlet-isolator flow characterization is that the measurements are discreet and a large gap existed between the readings. The global pressure map is not possible using transducers, and special geometrical allowances must be made for tubing. Inappropriately positioned pressure tubing would act as an obstacle to the main flow. The model to be used with a pressure transducer is usually very complex and would take longer to be manufactured.

Another more advanced method of measuring scramjet inlet-isolator performance is by utilising the “stream-thrust” concept. It was first proposed by Curran and Craig [10] and has since become the standard technique recommended by the scramjet textbook [11–13]. A practical example of this method can be found in Matthews *et al.* [14] and Matthews and Jones [15]. The concept relies on the conservation of mass, momentum and energy of the captured airstream of flow entering the inlet. The mass flow rate can be measured with a suitably designed mass flow meter, which would be attached at the inlet-isolator exit [16]. This would add to the intrusiveness of the flow and increases the probability of wind-tunnel unstart. The total drag of the model during a wind tunnel run could be measured with a force balance, but requires complex calculations to separate the drag contributed by the captured airstream from the skin friction drag on the cowl external surface and drag due to pressure tubings connected to the model [17]. The heat loss can be quantified by using a discreet thermocouple or a global surface temperature mapping technique. In essence, the stream-thrust measurement system is very complex and has large uncertainties contributed by each measurement technique. Thus, a new and better inlet-isolator performance measurement method is proposed by this article using luminescent measurement systems, which can provide global surface mapping of flow properties, whilst requiring a simpler setup.

Pressure- and temperature-sensitive paints (PSP and TSP) have been applied in the past to investigate the flow behaviour on the forebody of a scramjet inlet. Matsumura *et al.* [18] have shown that TSP can be employed to study the boundary layer instability and transitional characteristics in the presence of roughness elements on the forebody. Goertler and other streamwise vortices can be visualized easily with TSP. Yang *et al.* [19,20] has demonstrated the viability of quick response anodized aluminium PSP (AA-PSP) in investigating the unsteady behaviour of flow on a double-ramp scramjet forebody. The dynamic response of the pressure detected by the AA-PSP system matched the response of Kulite transducers. Global surface measurement of the internal flow inside the isolator section has been shown by H*ä*berle and G*ü*lhan [4,21] and Gruhn and G*ü*lhan [7]. They employed the infrared thermography (IR) technique to map the Stanton number distribution on the isolator sidewall. The heat transfer pattern from the IR map corresponded to the internal shock structures observed by schlieren images. Recently, Che Idris *et al.* [22,23] have applied PSP on the internal isolator surface, and the pressure profile showed that the spatial gap between the closely positioned pressure transducer could contain vital flow information.

The current study investigates the external and internal flow characteristics of a generic two-dimensional scramjet inlet-isolator using PSP techniques. An angle-of-attack (AoA) range from 0° to 4° is examined. The pressure maps are accompanied by colour schlieren visualization methods for a deeper understanding of the flow structures. Pressure patterns on the sidewall surface made by the glancing shock train are taken as the basis for calculating the isolator exit Mach number.

## 2. Scramjet Inlet-Isolator Performance

Scramjet inlet-isolator performance is classified according to its total pressure ratio and kinetic energy efficiency. Both performance indicators assume a quasi one-dimensional streamtube of flow through the inlet, such as depicted in Figure 1.

In Figure 1, Station **0** is the captured freestream prior to compression. Station **3** is downstream of the internal compression region and connects the inlet-isolator with the combustor. Flow properties at Station **3** are used as input for all performance calculations.

### 2.1. Total Pressure Efficiency, *π_c_*

Total pressure efficiency, *π_c_*, is defined as the ratio between the stagnation pressure in Station **3** and Station **0**. It demonstrates the loss in total pressure associated with the compression process [11–13]. As compression in the scramjet inlet is usually done by a discrete number of shocks, *π_c_* indicates the sum of loss due to each shock. Total pressure efficiency is also heavily influenced by shock wave-boundary layer interactions and, to a smaller extent, by the viscous loss as the flow stagnates due to the no-slip condition at the wall surface. To calculate *π_c_*, Equation (1) is applied:

(1) $$\pi_{c}=\frac{p_{t3}}{p_{t0}}$$

### 2.2. Kinetic Energy Efficiency, η_KE(ad)_

Kinetic energy efficiency, *η_KE_*_(_*_ad_*_)_ is defined as the ratio of kinetic energy contained in the flow at Station 3, if it were to be expanded isentropically to freestream pressure, to the kinetic energy available initially in the freestream flow [11–13]. It measures the efficiency of the compression process in terms of energy management. At hypersonic speeds, the freestream flow already contains a significant amount of kinetic energy, and the purpose of a scramjet engine is to produce thrust by increasing the flow velocity exiting the nozzle. If a significant amount of kinetic energy is lost during the compression process, then obviously, the thrust produced would be lower than at the ideal conditions. The equation for estimating *η_KE_* is given by:

(2) $$\eta_{KE\left(ad\right)}=1-\left(\frac{2}{\gamma -1}\right)\left(\frac{1}{M_{0}^{2}}\right)\left[\left(\frac{T_{x}}{T_{0}}\right)-1\right]$$

where:

$$T_{x}=T_{t3}{\left(\frac{p_{0}}{p_{t3}}\right)}^{\frac{\gamma -1}{\gamma }}$$

### 2.3. Compression Process Efficiency, η_C(ad)_

The final specific impulse and overall engine efficiency depends heavily on the overall adiabatic compression process efficiency, *η_C_*_(_*_ad_*_)_. This value indicates how much energy was spent by the compression process. It can be calculated as the ratio of total energy contained at Station 3 relative to the initial energy in the captured air volume at freestream [11]. Fortunately, this value is linearly related to *η_KE_***_(_***_ad_***_)_**:

(3) $$\eta_{C\left(ad\right)}=1-\frac{\left(\gamma -1\right)M_{0}^{2}}{2}\left(\frac{1-\eta_{KE\left(ad\right)}}{\frac{T_{3}}{T_{0}}-1}\right)$$

## 3. Experimental Setup

### 3.1. High Supersonic Tunnel

**Figure 2.**
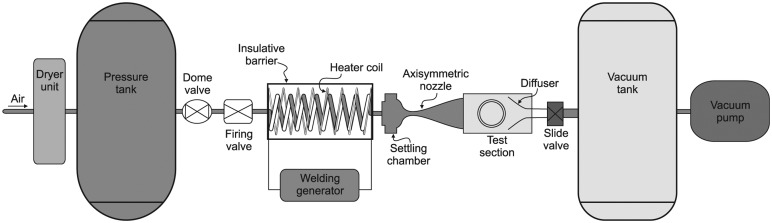
High supersonic tunnel (HSST) setup.

The experiments were carried out in a high supersonic tunnel (HSST) with the various components depicted in Figure 2. It is of an intermittent type and uses dry air as the working fluid, which is stored in a pressure reservoir at 16 bar. An electric resistive heater was used to raise the air temperature to avoid liquefaction during its expansion in the nozzle, up to 700 K at maximum enthalpy conditions. The tunnel can be fitted with different axisymmetric nozzles to produce Mach numbers between 4 and 6. The stagnation pressure can be set between 6 to 8 bar to vary the Reynolds number between 4.5 × 10^6^ m^−1^ and 15 × 10^6^ m^−1^ [24]. Typically, the useful tunnel running time is about 7.5 s [25].

For the current experiment, a 152-mm internal diameter nozzle capable of achieving Mach 5 flow was chosen. The Reynolds number was set to 13.2× 10^6^ m^−1^ by setting the stagnation pressure and temperature to be 6.5 bar (± 0.05 bar) and 375 K (± 5 K), respectively. Thus, the stagnation enthalpy is about 3.7 × 10^5^ J/kg. The variation in the Mach number was approximately ± 0.4%, while the Reynolds number error was between ± 3.9%. The dimensions of the test section are 325 mm × 325 mm × 900 mm (height × width × length). There are two circular quartz windows 195 mm in diameter on either side of the test section for optical access. Further information about the wind tunnel facility is provided by Erdem and Kontis [24] and Erdem *et al.* [26].

### 3.2. Scramjet Inlet-Isolator Design

A generic inlet was specifically designed for the current experimental investigations. A two-dimensional inlet geometry was chosen to simplify the design process. The inlet forebody utilised a double-ramp shape to avoid unnecessary length and weight incurred if an isentropic compression surface is used [12]. Typically, an overall compression efficiency of about 0.9 could be achieved with a 3-shock inlet system [27].

Achieving a shock-on-lip (SoL) condition in inviscid Mach 5 flow was set as the main design driver. The inlet was designed to have almost an equal compression shock strength in order to maximize the total pressure recovery [28]. Another design constraint that dictates the design process is that the ratio of the Mach number at the combustor face to the Mach number at freestream must not be smaller than 0.38 to avoid excessive temperature, which would produce the dissociation of air [27]. The ideal condition of shock-on-shoulder (SoS) was neglected, and the cowl shock was deliberately designed to impinge downstream of the shoulder in order to satisfy the Kantrowitz limit [29]. Satisfying the Kantrowitz limit is necessary to ensure that the inlet would self start.

**Figure 3.**
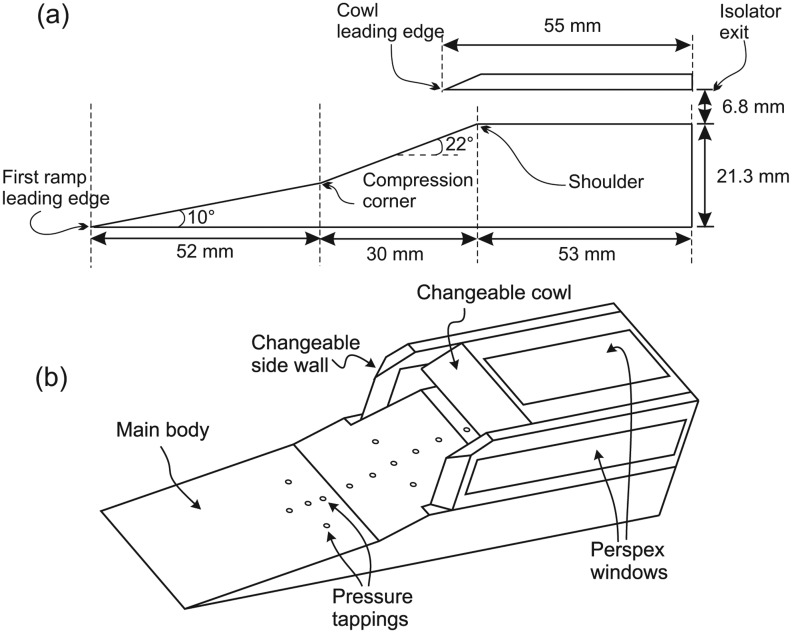
Basic dimension of the generic scramjet inlet-isolator.

**Figure 4.**
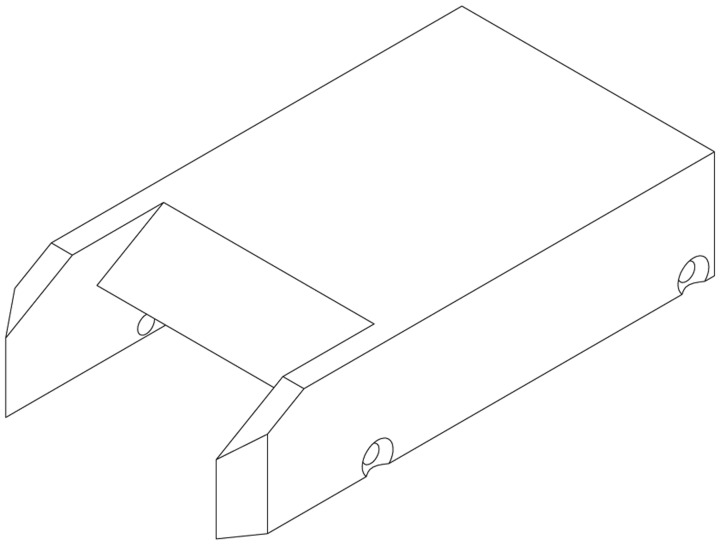
Single-piece cowl components made entirely from quartz to allow for an unobstructed view of the isolator surface.

The resulting design is shown in Figure 3a. It has a total length of 155 mm with an isolator height of 6.8 mm. The width of the inlet is 36 mm. The sidewalls were equipped with Perspex windows secured by an aluminium frame, such as shown in Figure 3b. For full viewing access to the isolator surface without any obstruction, a solid cowl component made from quartz was used (see Figure 4). Twelve pressure tappings are distributed alongside the model centerline and connected to Kulite pressure transducers.

### 3.3. Flow Diagnostics

Surface static pressures along the centreline of the model were measured using Kulite*^®^* (XTE-190 M, 0–70 kPa) pressure transducers. Typically, the uncertainty is about ±3%. The stagnation pressure and temperature were measured using Kulite (XTE-190M, 0–100 psi) (Kulite Semiconductor Products Inc., Leonia, NJ, USA; and a K-type thermocouple, respectively. A National Instrument (NI*^®^* PCI-6251 Data Acquisition (DAQ) (National Instruments Inc., Austin, TX, USA) card was used to record the analog signals after they were conditioned by a SXCI-1000 unit. The measurement trigger and control of the sampling rate was provided by Labview*^®^* v8.5 software (National Instruments Inc.). The sampling rate was set at 30 kHz to capture any unsteadiness in the flow.

### 3.4. Colour Schlieren

**Figure 5.**
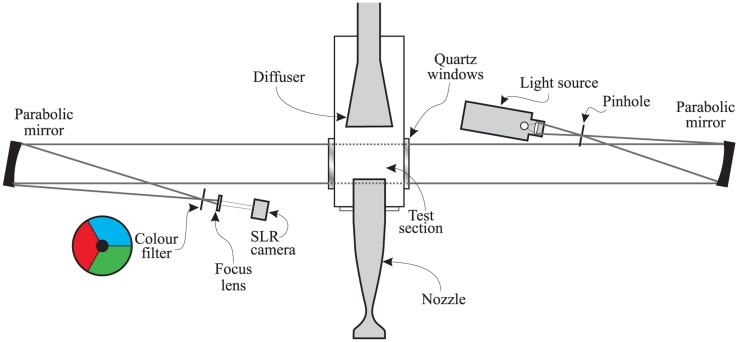
Colour schlieren setup.

Colour schlieren has been adopted as the main visualisation technique. Settles [30] argued that colour schlieren images provided more information, as they are more evenly illuminated compared to black and white images. Flow features are easier to identify in colour, as the technique balances the requirements of high sensitivity and a broad measurement range. The other advantage offered by a colour schlieren system is that it can measure the direction of density gradients [31]. The schlieren system used in this study is adopted from Yang *et al.* [19,20] and shown in Figure 5. It is of Toepler's z-type and consisted of a 300 W, continuous xenon arc lamp, a focusing lens, a circular pinhole, two parabolic mirrors, a tri-coloured knife edge and a set of Hoya 49-mm lenses for image focusing. The parabolic mirrors have a 5-degree offset angle relative to their axis in order to avoid astigmatism and coma. The second mirror reflects the parallel beam passing through the tunnel test section towards the camera. A tri-coloured knife edge filter with a black spot in the middle is located at the focus point between the second mirror and the camera. A Canon EOS-450D (Canon Inc., Tokyo, Japan) was used for schlieren image acquisition. It is capable of capturing 12 megapixels images and was set to continuous shooting mode of 3.5 frames per second with a shutter speed of 1/4,000 s.

### 3.5. Pressure-Sensitive Paint (PSP)

PSP consisted mainly of oxygen-sensitive luminescent molecules and a binder that are mixed together to form paint. For flow diagnostics applications, photons are shone onto the molecules in order to excite them to a higher energy state. Excited molecules would then emit photons of a longer wavelength, as they return to the ground energy state. In the presence of oxygen molecules, some of the excited energy is quenched by oxygen molecules, thus reducing the energy radiated by the excited molecules. As the oxygen concentration is linearly proportional to static pressure, the intensity of photons radiated would then be inversely proportional to static pressure [32]. A detailed explanation on the luminescent kinetics principle of PSP can be found in the literature [32,33].

The preparation of PSP for this current experiment follows the example from [34,35] with some modifications. Methyl triethoxysilane (MTEOS) was chosen as the sol-gel polymer binder and was mixed with ethanol and hydrochloric acid (HCl). Ethanol acts as the solvent, and HCl would assist in the chemical reaction as the catalyst. The high pressure sensitivity luminophore of platinum-tetrakis(pentafluorophenyl)porphyrin (PtTFPP) molecules were added to the solvent mixture with a concentration of 4 mM ± 0.2 mM. The final mixture was treated with 30 minutes of ultrasonic mixing to ensure that all the luminophore particles were dissolved.

The scramjet inlet forebody, isolator and sidewall surface were polished prior to the application of the base paint. A white base coat was painted to act as a reflector to direct emitted photons towards the detector. The luminophore-polymer mixture was painted afterwards with up to 20 layers of thin coats. The paint was left to dry after every two coatings to ensure that the overall coatings would not be easily peeled. After the final layer was applied, the model was left in an oven for 7 h at temperature of 343 K to ensure all the solvent was evaporated.

**Figure 6.**
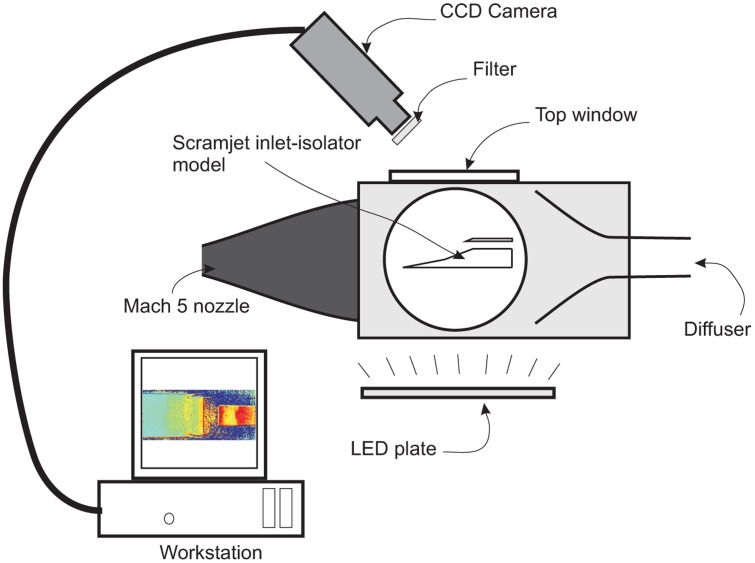
Side view of the pressure-sensitive paint (PSP) Setup 1 system configurations, where the pressure map on the isolator surface was recorded by placing the camera on the top of the test section. Note that the LED is normal to the test section's side windows and not underneath.

Two PSP system configurations were used for this study. The first, as depicted in Figure 6, captured the pressure intensity map on the compression ramps and isolator surface. The camera was positioned on top of the wind tunnel. The illumination source for this setup was provided by two LED (Light Emitting Diode) panels. Discreet pressure readings for i*n situ* calibration were provided by the Kulite pressure transducers, where the measurements were taken at the same time as the intensity map was recorded by the camera.

**Figure 7.**
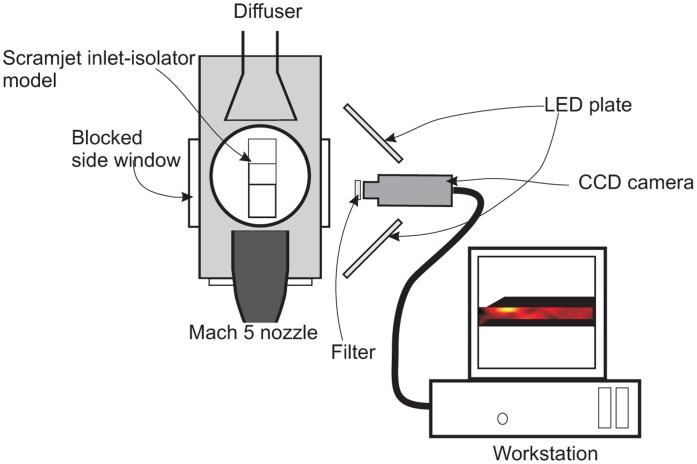
Plan view of the PSP Setup 2 system configurations, where the pressure map on the isolator sidewall was recorded by placing the camera on the side of the test section.

PSP system Configuration 2, shown in Figure 7, was utilized for the measurement of the pressure map on the isolator sidewall. The camera was positioned such that its capture plane was parallel to the wind tunnel side window plane. The two LED panels were fixed on the side of the camera to provide even illumination on the model. The isolator sidewall nearest to the camera was equipped with a Perspex window to provide viewing access, while the opposite sidewall, which was spray-painted with PSP, was made from aluminium. There were no pressure taps on the sidewall; thus, its pressure intensity was calibrated against the measured pressure, which resulted from PSP Setup 1.

The LED panels consisted of 192 unit of a UV5TZ-395-30 model LED with a peak wavelength of 395 nm. This peak wavelength coincides with the peak excitation wavelength of the PtTFPP luminophore [34]. A LaVision^®^ Imager Intense (12-bit CCD (Charge-coupled Device)) camera (LaVision Inc., Gottingen, Germany) was used for image capture for both PSP experimental setups. The exposure time was set at 7.5 ms with a capture rate of 10 Hz. The camera lense was fitted with a 530-nm long-pass and a 700-nm cut-off filter. Davis^®^ 7.0 software (LaVision Inc.) was utilised to control the camera and for viewing real-time intensity change during the experiments. The MATLAB^®^ image (The MathWorks Inc., Natick, MA, USA) processing toolbox was then used for data processing. The camera was set to capture a series of 100 images for every wind tunnel run. To improve the signal-to-noise ratio of the recorded pressure intensity, a final 30 images before the end of each wind tunnel run were summed together. The signal-to-noise ratio is proportional to the square root of the number of images [33]. Using image ratios ensures that any effects of non-uniform image illumination or paint distribution are eliminated.

Temperature sensitivity has always been a setback with PSP experiments. One way to minimize the temperature effects is by using *in situ* calibration, which was indeed used in the experiment described in this paper. The *in situ* calibration relates the luminescence intensity during the test with the corresponding pressure at the specific location with specific temperature. It is believed that the combination of using *in situ* calibration and the fact that the model is made from aluminium would therefore result in having a high thermal conductivity, ensuring a fairly uniform temperature distribution. Another factor is the short duration of the test time, which would also minimize temperature effects over the model surface.

To minimized error due to the temperature sensitivity of the paint, reference wind-off images were taken immediately after each wind tunnel run [36]. Reference wind-off images were needed for *in situ* calibration and consisted of intensity maps at a known pressure without any flow passing the model. Both wind-on and wind-off images must be corrected for unplanned photon detection from the surroundings. This can be done by subtracting dark images captured as the illumination light source and wind-tunnel were turned off.

**Figure 8.**
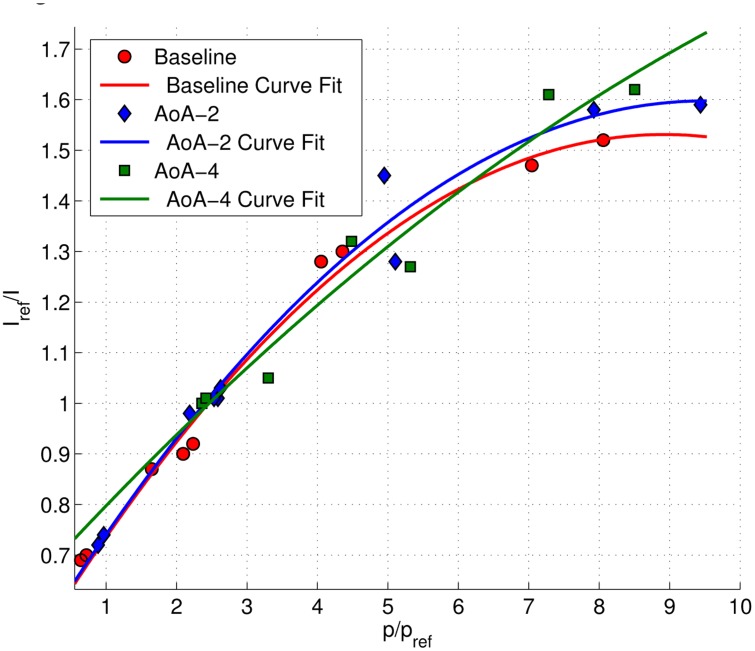
The Stern–Volmer calibration plot for the isolator surface pressure using discreet pressure readings from Kulite.

**Figure 9.**
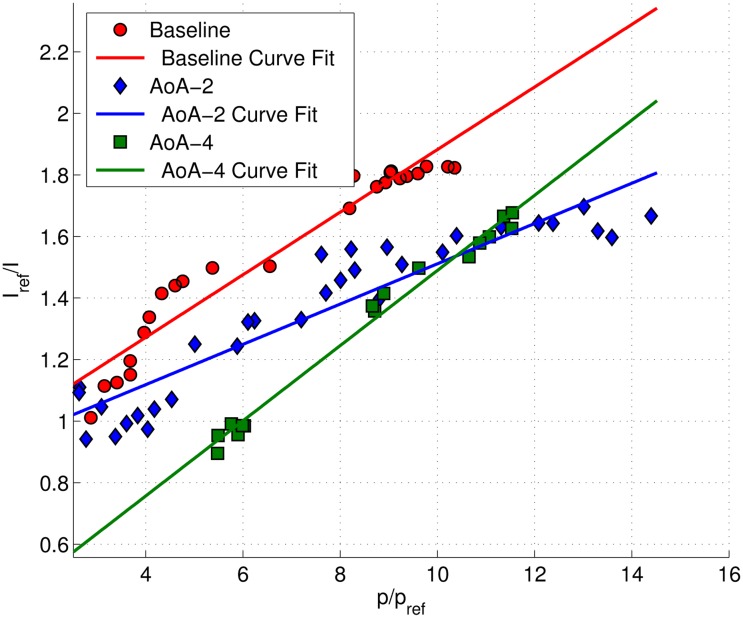
Stern-Volmer calibration plot for sidewall pressure using pressure profile readings obtained on the isolator surface. AoA, angle-of-attack.

The calibration plot of the forebody and isolator section are presented in Figure 8. The Stern–Volmer plots were in the form of quadratic equations, as discreet pressure readings were taken from two regions (*i.e.*, forebody and isolator), which has a significant difference in their average flow temperatures. Since *in situ* calibration can be significantly affected by temperature, it would be inappropriate to assume a linear calibration curve to relate the luminescence intensity to the static pressure over two regions with large temperature variance. The calibration process was repeated for each angle of attack case. The calibration curve for the isolator sidewall pressure map utilized only the linear curve and is shown in Figure 9. The intensities from the sidewall were related to the static pressure readings produced by the PSP map on isolator, as there were no pressure tappings on the sidewall. A linear curve fit was considered, since the region considered was limited to the isolator section only. Calibration was repeated for each angle of attack case.

## 4. Numerical Analysis

Van Wie and Ault [37] advocated the combined method of experimental and numerical investigation for scramjet inlet performance measurement. Validated numerical calculations would provide comprehensive flow field data and can avoid the need for intrusive measurement techniques inside a small inlet model [12]. For this research, Favre averaged Navier-Stoke (FANS) equations are used to model the inlet in the two-dimensional environment and are then solved using commercially available software FLUENT^®^ (ANSYS Inc., Cecil Township, PA, USA). Second order accuracy is achieved by utilising the second order spatially accurate upwind scheme (SOU) with Roe's flux-difference splitting.

The shear-stress transport (SST) *κ-ω* model of Menter is adopted to simulate turbulent flow. This turbulence model has the combined advantage of *κ-ω* at near the wall region and *κ-ϵ* at the inviscid core flow region outside the boundary layer. The flow would normally be laminar unless tripped [25,38], but the flow separation expected on the compression corner would induce a transition to turbulence [22]. Hence, the turbulence intensity at freestream was set to 0.5% to allow for correction for transition. The turbulence viscosity ratio is fixed as one. Stability is ensured by setting the Courant-Friedrichs-Levy (CFL) number to 0.5 initially and gradually increasing it by the same value every 1,000 iterations. Inviscid solutions for every parametric case are inputted as the initialisation conditions for turbulence computation by the solver.

**Figure 10.**
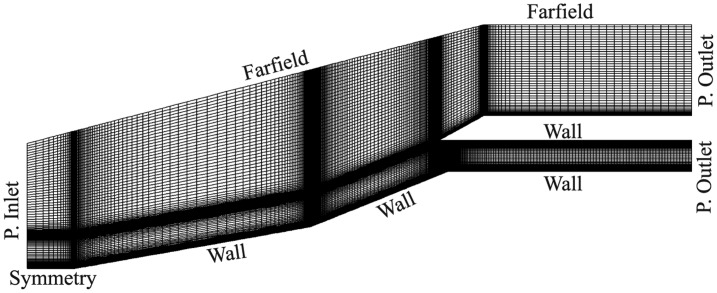
Medium mesh for the baseline case.

**Figure 11.**
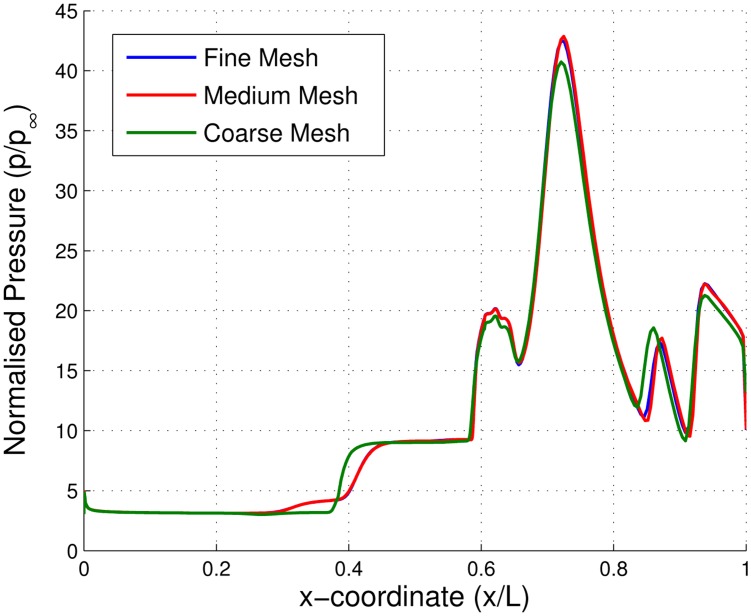
Normalized static pressure along the centerline for the baseline scramjet inlet-isolator model using different mesh grid densities.

The computational domain is bounded by a pressure inlet, symmetry, far-field, constant temperature walls and two pressure outlets (see Figure 10). The properties for the pressure inlet and far-field are taken from HSST flow conditions. The symmetry region between the pressure inlet boundary and the first compression ramp wall helps to maintain stable iterations. Properties at the two pressure outlets were calculated assuming free flow, where the flow would exit the isolator, expanding to freestream conditions. The mesh for the baseline, AoA-2 and AoA-4 cases were all made using quadrilateral cells with a higher density grid concentrated around a large flow turning region. The first cell height was set to two microns in order to achieve a *y*^+^ value of one. The sensitivity of the results to grid density was analyzed by using three different grid refinement levels. The mass flow rate balance between mass coming in and going out of the computational domain of less than 1% is considered as the main convergent criterion along with the stability in other residuals. It was found from Figure 11 that a medium grid of 52,250 has a very close fit with the pressure readings from a fine grid of 73,840 cells. A coarse grid of 34,485 cells also showed close approximation with the other two grid levels. Therefore, the medium grid level is adopted for all three parametric cases.

Flow field data at the exit of the isolator were reduced using the mass averaging technique. This is to avoid unreal entropy increase associated with mixing if other averaging techniques are used [12]. The averaged values were then input into a set of equations to calculate *π_c_* and *η_KE_*. Numerical schlieren images are produced by Tecplot 360^®^ software (Tecplot Inc., Washington, DC, USA) by computing the density gradient in the *y*-direction for the whole flow field. The density gradient distribution are then visualised using a colour mask to mimic the experimental colour schlieren.

## 5. Results and Discussions

### 5.1. Internal Shock Structures

**Figure 12.**
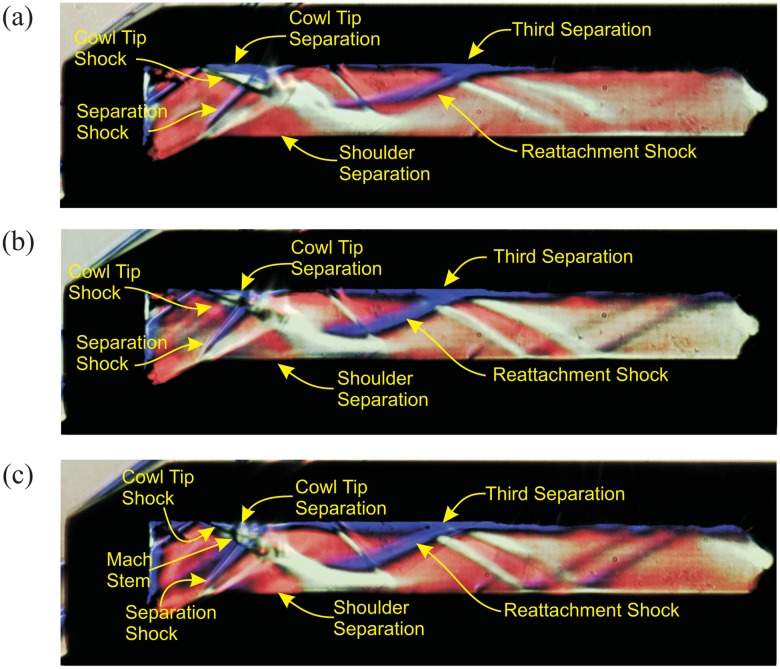
Comparison of schlieren images for different cases: (a) baseline; (b) AoA-2; (c) AoA-4.

Experimental schlieren images for all angle of attack cases, focusing only on the throat and the isolator exit, are presented in Figure 12. For the baseline case, the shocks from the external compression ramp impinge very close to the cowl tip, thus enabling the inlet to capture a near maximum mass flow rate. This observation demonstrates the minimum boundary layer displacement on the compression ramp. Type 5 off-design wave interactions [39] form at the cowl tip region for the baseline case (see Figure 12a). This type of interaction is defined as the shock structure produced by the interaction between the cowl shock and the shoulder expansion wave [39]. The entire isolator section contains three boundary layer separation bubbles, which obstruct the core flow and negatively affect its performance. Each of the separation bubble is identified by the presence of separation and re-attachment shocks. The reflections of shocks from the cowl tip and the separation shock form a shock pattern, which is called the “background wave” [40]. The background wave is defined as the internal shock structure in the isolator section that formed due to reflections of uncancelled cowl shock. This background-wave is different from a shock train, as there is no back pressure acting at the isolator exit, but it acts as a precursor for their formation [40]. Since the separation originated just upstream of the throat, the introduction of backpressure at the isolator exit could easily unstart the inlet [41]. The unstart mechanism related to the shoulder separation bubble has been observed by Tan *et al.* [42], where in his model, unsustainable backpressure at the isolator exit forced the separation at the throat to oscillate by expanding and shrinking periodically. The shoulder separation in the figure induces two more separations via the boundary layer interactions at the impingement locations of its separation and re-attachment shock. The combined effect of cowl surface separation and shoulder separation reduces the effective isolator entrance height by approximately 70%. This observation shows the importance of mitigating the problem of boundary layer separation at the throat region of a scramjet inlet.

With the introduction of AoA at the windward side, the cowl tip separation can be seen to decrease in size, which is shown in Figure 12b,c, for AoA = 2° and 4°, respectively. Shocks from the compression ramp move upstream from the cowl tip, making it more resistant to separation.

An interesting shock formation can be observed experimentally for the AoA = 4° case in Figure 12c. A Mach stem forms at the intersection between the cowl tip shock and the shoulder separation shock, inducing a large stagnation pressure loss. The mechanism for this shock formation is that the Mach number entering the isolator decreases with every increase in AoA, but the flow turning angle imposed onto the flow stays constant at 22°. This flow turning angle is unsustainable for the flow entering the isolator for the AoA = 4° case, thus forcing it to decelerate to subsonic speed via a Mach stem. Such a shock-shock interaction is called Edneys Type II [43]. This subsonic pocket forms locally and does not spread towards the isolator exit, as oblique shock reflections could be observed downstream.

### 5.2. Centerline Pressure Profiles

**Figure 13.**
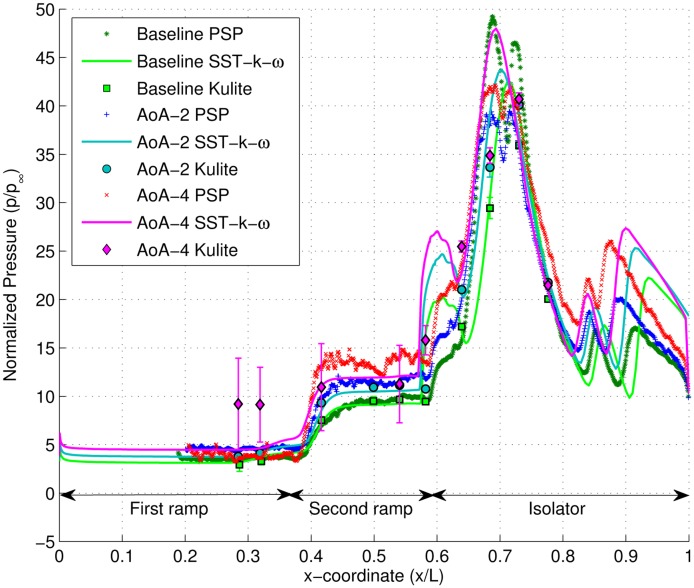
Normalized static pressure along the centerline of scramjet inlet model for different cases: (**a**) baseline; (**b**) AoA-2; (**c**) AoA-4. SST, supersonic tunnel.

The pressure profile along the centreline of the model, given in Figure 13, provides a better understanding of shock structures inside the isolator section. Since the background wave inside the isolator formed due to the impingement of the cowl tip shock downstream of the shoulder, pressure profiles provided vital indications on the distortion level [37]. Peak pressures, crucial for structural integrity considerations, are provided by the continuous PSP pressure. Due to manufacturing constraint, the isolator section could only be fitted with four pressure tappings covering only half of the isolator length. The application of PSP provided important pressure trends on the other half of the isolator section.

CFD (Computational Fluid Dynamics) generated pressure profiles are included in Figure 13 for comparison. Good agreements can be observed between PSP, CFD and Kulite pressure readings for all cases, especially on the first ramp. On the second ramp, subtle difference can be detected between the pressure readings from the three sources. PSP pressure profiles on the second ramp tend to have a slightly higher magnitude compared to Kulite and CFD predictions.

There is significant uncertainty in Kulite pressure readings for the AoA = 4° case, especially on the first and second ramp surfaces. The static pressure at these regions has violence fluctuations of magnitude, which produce a large error bar when the pressure is averaged. Each error bar represents the standard deviation of pressure at their respective locations. The root cause of the pressure fluctuations is thought to be the spatial oscillations of the compression corner separation shock.

At the isolator entrance, CFD tends to over predict the strength of the shoulder separation shock. The locations of the separation onset are also shown to be slightly more upstream by the numerical analysis. Inside the isolator section, generally, there are three pressure peaks marking: (1) the re-attachment point of the shoulder separation bubble; (2) the impingement points of separation; and (3) the re-attachment shock from the third separation bubble. The three pressure measurement methods differ quite significantly in predicting the location and magnitude of the shock impingement from the third separation bubble re-attachments.

### 5.3. Flow Three-Dimensionality

**Figure 14.**
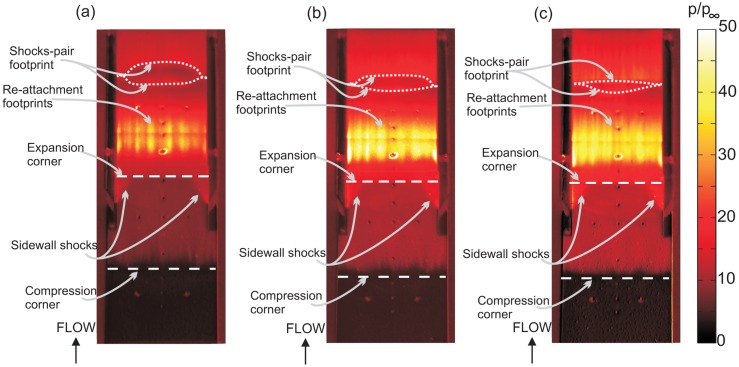
Normalized static pressure map taken from the top of the model.

The three-dimensionality of the flow over the external compression ramp and inside the isolator section was investigated using a global surface pressure map on the surface and shown in Figure 14. Overall, the pressure patterns are similar between the three angles of attack. Streaks on the second ramp, just downstream of the compression corner, are present for all cases, indicating the formation of streamwise vortices. Sidewall shocks can be seen glancing from the leading edge of the isolator sidewalls. The sidewall shock angle remains roughly similar, even though the flow on the second ramp has a lower Mach number with increasing AoA. The sidewall contribution to the overall compression is thought to be minimal, since the sidewall shocks does not extend downstream into the isolator. A very high pressure belt can be observed at the re-attachment line of the shoulder separation bubble. In all cases, shock-pair footprints can be detected further downstream, marking the impingement lines of separation and the re-attachment shocks from the third separation bubble. The pair of separation and re-attachment shock footprints are symmetrically straight without a significant curve, except at region near the sidewall, where they coalesced together. It is speculated that the third separation bubble diminishes near the sidewall for all AoA cases.

**Figure 15.**
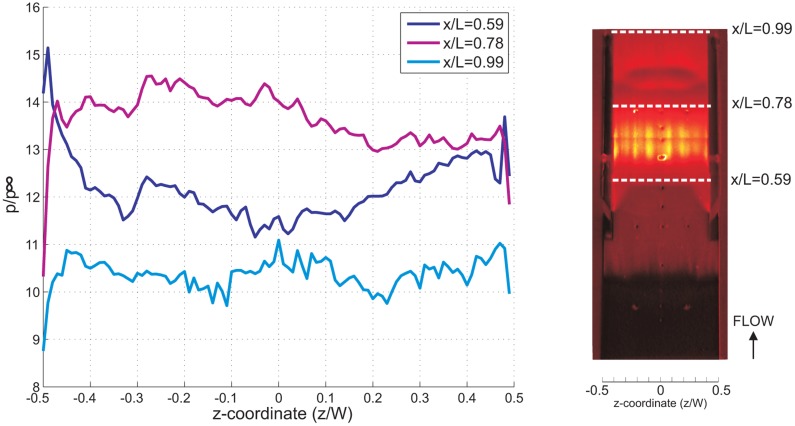
Normalized spanwise static pressure for the baseline case taken at the isolator entrance *x*/*L* = 0.59, the middle of the isolator *x*/*L* = 0.78 and the isolator exit *x*/*L* = 0.99.

**Figure 16.**
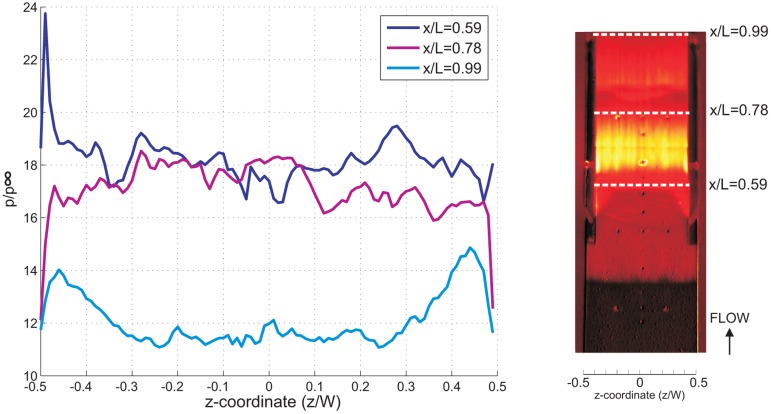
Normalized spanwise static pressure for the AoA-2 case taken at the isolator entrance *x*/*L* = 0.59, the middle of the isolator *x*/*L* = 0.78 and the isolator exit *x*/*L* = 0.99.

**Figure 17.**
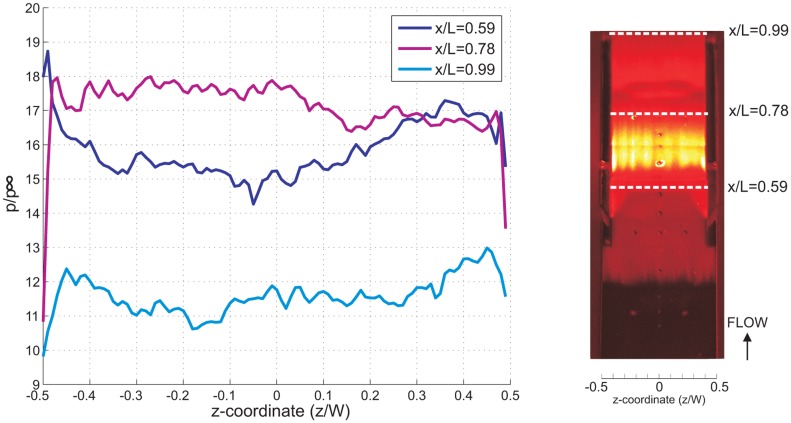
Normalized spanwise static pressure for the AoA-4 case taken at the isolator entrance *x*/*L* = 0.59, the middle of the isolator *x*/*L* = 0.78 and the isolator exit *x*/*L* = 0.99.

The spanwise pressure distribution at the isolator entrance (normalized *x*-coordinate, *x*/*L* = 0.59), mid-point (*x*/*L* = 0.78) and isolator exit (*x*/*L* = 0.99) for all cases has been plotted and compared in Figures 15, 16 and 17. The spanwise non-uniformity shows the extent of the three-dimensionality effects of the flow inside the isolator. Generally, for all cases, and for all stations, almost-periodic spanwise pressure fluctuations are observed, which can be attributed to the manufacturing finish on the quartz cowl component. The accuracy of the spanwise readings are thought not to be significantly affected, and the error due to the quartz manufacturing finish is estimated to be approximately 2%. It can also be said that the three-dimensional effect was very significant in the 5% of the total spanwise length from the sidewall. This is true, except at the isolator exit for the AoA = 4°case, where the effect extends to 20% of the total spanwise length from the sidewall, indicating a significant three-dimensional flow effect.

The average pressure and standard deviation for all cases at three stations (taken from Figures 15, 16 and 17) are compared together in Table 1. Typically, the standard deviation in spanwise pressure is lower than 5.5%, which allows for the assumption of two-dimensional flow throughout the isolator. This is true, except for the AoA = 4°case, where at the exit of the isolator, the standard deviation in spanwise pressure is 8.1%. There is a general trend of an increasing three-dimensionality effect at the isolator exit for increasing angle of attack. This suggests that computational simulation of a scramjet inlet-isolator for a high angle of attack must be done in the three-dimension domain to ensure better results.

### 5.4. Prediction of Flow Properties and Inlet-Isolator Performance

**Figure 18.**
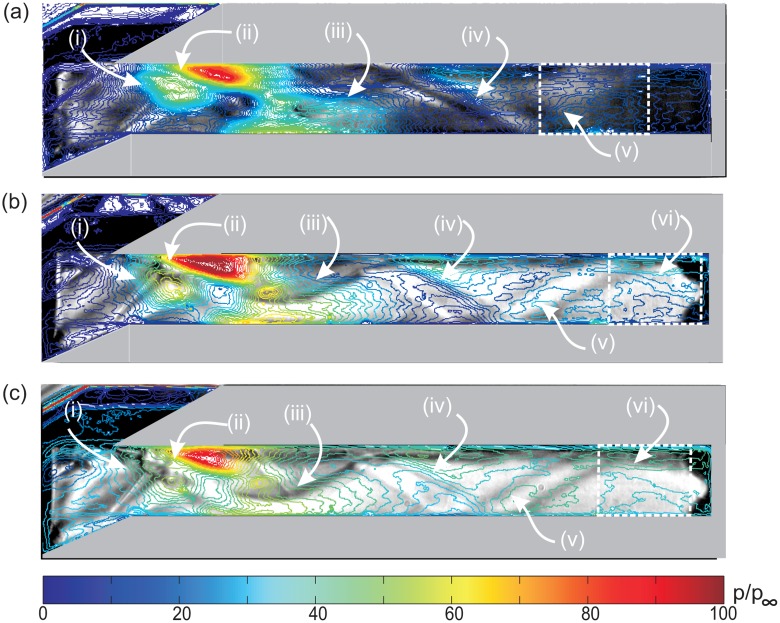
Normalized pressure contour on the isolator sidewall surface layered on top of the grey scale schlieren image for (**a**) baseline; (**b**) AoA-2; and (**c**) AoA-4.

The pressure contour on the isolator sidewall for all three cases is compared in Figure 18. The PSP maps on the sidewall are calibrated with their corresponding pressure profile obtained using PSP Setup 1. All pressure maps were normalized to freestream pressure. A grey scale schlieren image is layered underneath the sidewall pressure contour to provide more information and easier identification of the flow phenomena.

Glancing shocks can be identified by contour lines of increasing magnitude, which coalesced closely together. It is observed that glancing shock patterns on the sidewall for all cases do not follow exactly the shock patterns detected by schlieren. This is due to the fact that the schlieren image effectively integrates the flow field along the spanwise direction, whilst the pressure contour in the figure visualizes the flow touching the sidewall only. If the isolator flow field is truly two-dimensional, the glancing shock patterns on the sidewall would be similar to the shock patterns recorded by the schlieren technique.

For the baseline case, in Figure 18a, glancing shock (**i**) is the cowl tip shock. Glancing shock (**ii**), found further downstream, is thought to be related to the re-attachment of cowl tip separation. The magnitude of this shock is very severe at about 100 times the freestream pressure. The elimination of the shoulder separation, which causes the cowl tip separation, would effectively solve this problem. Ideally, if the inlet achieved the shock-on-shoulder condition, where no background wave occurs inside the isolator, the structure of the cowl and isolator surface would be safe from such severe aerothermal loads. The glancing of re-attachment shock from the shoulder separation bubble was thought to be related to shock (**iii**). Interestingly, the separation shock from the shoulder separation bubble was not detected by the pressure contour. Similarly, there was only one glancing shock originating from the third separation bubble, labelled shock (**iv**) in the figure. This shows that the third separation bubble diminished at the sidewall, as discussed previously in Section 5.3. The reflection of shock (**iv**) was identified as the cause for glancing shock (**v**). This is the final shock detected by the pressure contour and has a lower pressure gradient in comparison to other shocks. This weak shock was assumed to be approaching the Mach wave limit, thus exerting only a minimal flow turning angle onto the flow. This assumption is important, as it allows us to consider the control volume surrounding the final shock, (**v**), for Mach number calculations without first needing to guess the initial flow direction angle upstream of the shock. This assumption is justified, since the formation of the internal shock patterns is only necessary for the isolator to re-align the flow direction, and with long enough of an isolator, the shock structure will diminish at the isolator exit. This assumption will be proven correct by calculating the turning angle the shock, (**v**), imposed onto the flow.

**Figure 19.**
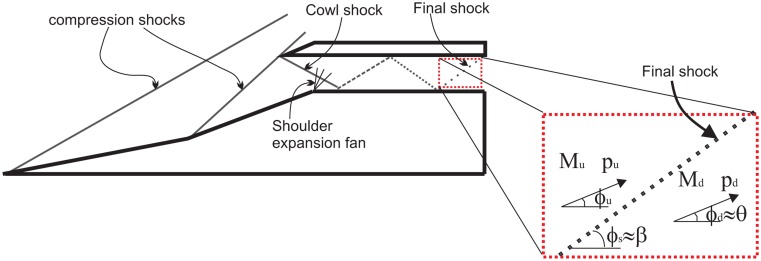
Schematic of the typical internal shock structure resulting from interactions between the cowl shock and expansion wave.

The shock structure in Figure 18a could be simplified into Figure 19. The sketch shows that the properties of the flow upstream and downstream of the shock can be related by simple oblique shock relations given by:

(4) $$\frac{p_{d}}{p_{u}}=1+\frac{2\gamma }{\gamma +1}\left[M_{u}^{2}\sin^{2}\beta -1\right]$$

(5) $$M_{d}=\frac{1}{\sin\left(\beta -\theta \right)}\sqrt{\frac{1+\frac{\gamma -1}{2}M_{u}^{2}\sin^{2}\beta }{\left(\gamma M_{u}^{2}\sin^{2}\beta \right)-\frac{\gamma -1}{2}}}$$

(6) $$\theta =\phi_{d}-\phi_{u}=\tan^{-1}\left[2\cot\beta \frac{M_{u}^{2}\sin^{2}\beta -1}{M_{u}^{2}\left(\gamma +\cos2\beta \right)+2}\right]$$

In Equations (4)–(6), *β* and *θ* are the shock angle and flow turning angle, respectively They are both referenced to the flow direction upstream of the shock, *ϕ_u_*, which is unknown. It is easier to measure, using Figure 18a, the shock angle relative to the horizontal axis, *ϕ_s_*.

Thus, to relate the measured shock wave angle, *β*, to the actual shock wave angle, *ϕ_s_*, the equation below can be used:

(7) $$\beta =\phi_{s}-\phi_{u}$$

If we assume that the final shock is very weak and its behaviour was almost like a Mach wave, its upstream and downstream flow angle, *ϕ_u_* and *ϕ_d_*, would be parallel to the horizontal axis. Hence, *θ* ≈ *ϕ_d_* and *β* ≈ *ϕ_s_*.

From Figure 18, the final shock for all cases appeared to be slightly curved. Thus, the final shock angle was measured by using trigonometric calculation of the right angle triangle made by the oblique shock. From the figure, the final shock angle was estimated to be about *β* = 28.09°. On average, the ratio of the static pressure rise across the final shock is found to be *p_d_*/*p_u_* = 1.025. This very small pressure rise ratio across the shock was the first proof that validates our assumption of a very weak shock wave.

Solving Equations (4) and (6), it is found that the flow turning angle imposed by the final shock was *θ* = 0.42°. This is very much consistent with our assumption of a very weak shock wave. Further manipulating Equations (4) and (5), it is found that the Mach number downstream of the shock *M_d_* = 2.131. This is taken as representative of isolator exit Mach number *M*_3_. This magnitude is very close to the CFD predicted averaged exit Mach number for the baseline case, where *M*_3(CFD)_ = 2.088.

Oblique shock relations are not valid if the shock considered is actually a Mach wave, thus a check must be made to ensure that the final shock seen in Figure 18a is truly an oblique shock. The Mach angle, *μ*, can be determined using Equation (8):

(8) $$\mu =\text{arcsin}\left(\frac{1}{M_{u}}\right)$$

It was found that *μ* = 27.78°, and this was smaller than the shock angle measured from Figure 18a, thus allowing the shock to be analyzed with oblique shock relations. However, *μ* and *β* are very close in magnitude, showing that the final shock was indeed very weak. This also demonstrates that the isolator has enough length to allow the flow to achieve maximum uniformity.

The average static pressure at the sidewall trailing edge in Figure 18a was taken as representative of the average one-dimensionalized static pressure of the isolator exit, *p*_3_. By inserting the calculated *M*_3_ and *p*_3_ into the isentropic flow relations in Equation (9) below, the total pressure and static temperature of the flow at the isolator exit can be calculated:

(9) $$\frac{p_{3}}{p_{t3}}={\left(1+\frac{\gamma -1}{2}M_{3}^{2}\right)}^{\frac{-\gamma }{\gamma -1}}={\left(\frac{T_{3}}{T_{t3}}\right)}^{\frac{\gamma }{\gamma -1}}$$

The calculated flow properties allow for the calculations of *π_c_* using Equation (1), *η_KE_*_(_*_ad_*_)_ using Equation (2) and *η_C_*_(_*_ad_*_)_ using Equation (3). The only assumption is that the flow is adiabatic, thus allowing the simplifications of the calculations, since there is no loss in the stagnation temperature. This is the standard practice in scramjet inlet performance calculations [12]. However, this method will be able to produce more realistic flow property estimation if the temperature map contours were provided alongside the pressure map contours.

Extending similar analysis to the AoA = 2° and 4° cases, it is found that their shock patterns were similar to the baseline, except that they are more compact and contained extra glancing shock (vi) (see Figure 18b,c). They act as the final shock for Mach number estimation, and their shock angles are measured to be 28.97° and 34.35° for the AoA = 2° and 4° cases, respectively. The pressure ratio across the respective shocks were measured to be 1.075 and 1.016. The flow properties and performance indicators from all cases are compared together in Table 2:

The CFD predicted isolator exit Mach numbers for the AoA = 2° and 4° cases were 2.05 and 1.89. These values are very close to the values calculated experimentally, listed in Table 2. For the baseline and AoA = 2°, CFD predicts a lower isolator exit Mach number compared to the experiments. However, for the AoA = 4° case, the CFD predicted a higher value, since it did not resolve the occurrence of the Mach stem at the isolator entrance, such as observed experimentally.

The baseline case achieves modest, if not unsatisfactory, performance. Its compression ratio is less than 20, and at the same time, it wasted a significant amount of total pressure, with only 32% of it left after the compression process. Its kinetic energy efficiency is poor, with only 0.92, which is then translated into a very modest compression process efficiency of 0.82. Heiser and Pratt [11] suggested that for a typical three compression shock inlet, the maximum compression process efficiency was about 0.9. The reason for such substandard performance was thought to be caused by the presence of the three separation bubbles inside the isolator section. They promote high stagnation pressure and kinetic energy losses to the flow.

When a modest angle of attack is introduced to the freestream flow, the performance improves significantly. For example, for the AoA = 2° case, the isolator exit static pressure increases to almost 30 times the freestream. Higher compression will translate into more thrust to be produced by the propulsion unit. The increased compression achieved by the inlet-isolator in this case does not sacrifice vital total pressure and flow kinetic energy, which, in fact, increase in comparison with Baseline. The improvements in total pressure and kinetic energy efficiency encourage a significant gain in compression system efficiency, close to the theoretical maximum for such inlets. The improvements for the AoA = 2°case are due to the decrease in the cowl tip separation size.

Increasing the angle of attack by a further two degrees improved the performance slightly. This achieves a subtly higher compression system efficiency in comparison to the AoA = 2° case, but this improvement came with quite a dramatic reduction of the stagnation pressure and kinetic energy. The overall compression increases the static pressure exiting the isolator close to 50 kPa, which is the ideal pressure for the high cycle efficiency of a wind tunnel-scale scramjet engine [44]. The appearance of the Mach stem causes the unnecessary increment of the static temperature of about 30 K from the AoA = 2° case. This value is very significant in comparison to the the difference in the isolator exit temperature between AoA = 2° and baseline, which was only about 4 K. The elimination of the Mach stem from the AoA = 4° case is suspected to further increase the inlet-isolator performance further.

The accuracy of the technique corresponds to that of the pressure transducers used for the calibration, which is in the range of 200 Pa, depending on the absolute pressure. Calibration uncertainty also comes from the curve fitting of the data. With the large signal levels from the luminophore, the major limit to the precision of the pressure is the detector noise, which is 3%. The spatial resolution of the technique depends on the minimum pixel size, which can be resolved by the CCD camera. In the present study, it is equivalent to a square of a side length of 0.1 mm. To simplify, the error of the estimation is illustrated by measuring the difference between the PSP predicted pressure against those from Kulite and CFD. This is summarized in Table 3.

## 6. Conclusions

An optical luminescence flow diagnostics system has been developed using PSP to investigate the characteristics of a scramjet inlet-isolator. The generic shape inlet-isolator was expected to perform poorly, since no viscous effect was considered during its design process, and the PSP diagnostics system has been able to fully capture this effect. The PSP system visualized and measured the flow on the compression ramps, isolator surface and isolator sidewalls. Colour schlieren flow images are complemented by the pressure map provided by PSP. The three-dimensionality of the flow inside the isolator has never been studied prior to this current study.

The pressure contours on the isolator sidewall provided the means to calculate the representative one-dimensionalised average isolator exit flow properties to be used in performance assessments. This new concept of inlet performance measurement is free from the problems, such as flow obstruction and a complex setup, associated with stream thrust analysis and pitot rake measurements. The effort for rapid parametric study crucial for design iterations has been reduced tremendously. This method is better than using the pitot rake, since its pressure measurement is global (albeit only on the surface), and it has less sources of error in comparison to the stream-thrust analysis.

The same concept could be applied for other luminescence measurement techniques, such as temperature-sensitive paint, infrared thermography, background oriented schlieren and many others, as long as they can give out an accurate flow property map on the sidewall.

## Figures and Tables

**Table 1.** Average spanwise static pressure and its standard deviation as an indicator of spanwise uniformity.

**Case**	**Avg Pressure at** *x*/*L* = 0.59	**Avg Pressure at** *x*/*L* = 0.78	**Avg Pressure at** *x*/*L* = 0.99
Baseline	12.18 ± 5.4%	13.63 ± 4.3%	10.37 ± 3.2%
AoA-2	15.86 ± 5.1%	17.11 ± 5.2%	11.58 ± 4.6%
AoA-4	18.20 ± 4.8%	17.11 ± 5.8%	12.07 ± 8.1%

**Table 2.** The isolator exit flow properties and its associated performance indicators for different cases.

**Case**	*M*_3_	*p*_**3**_**(bar)**	*p_t_*_**3**_**(bar)**	*T*_**3**_**(K)**	*C_r_*	***π_**C**_***	*η_KE_*_**(**_*_ad_*_**)**_	*η_C_*_**(**_*_ad_*_**)**_
Baseline	2.31	0.22	2.07	194.92	17.7	0.32	0.92	0.82
AoA-2	2.09	0.33	2.98	198.65	27.2	0.46	0.95	0.89
AoA-4	1.78	0.47	2.62	228.10	38.7	0.41	0.94	0.89

**Table 3.** Error estimation of PSP against Kulite and CFD.

**Case**	**Standard Deviation between PSP and Kulite**	**Standard Deviation between PSP and CFD**
Baseline	6.04	5.97
AoA-2	1.61	2.45
AoA-4	2.10	3.13

## Nomenclature

*M*: Mach number
*p*: Pressure, Pa
*T*: Temperature, K
*β*: Shock wave angle relative to flow direction upstream of shock, degree
*δ*: Flow direction relative to horizontal axis, degree
*ϕ*: Shock or flow direction angle relative to horizontal axis, degree
*γ*: Air specific heat ratio
*μ*: Mach angle, degree
*η_C_*_(_*_ad_*_)_: Compression process efficiency
*η_KE_*_(_*_ad_*_)_: Kinetic energy efficiency
*π_c_*: Total pressure efficiency
*θ*: Flow turning angle relative to initial flow direction, degree

**Subscript**
*d*: Downstream of a shock wave
*I*: Intensity level of PSP
*L*: Total length of the scramjet inlet-isolator model, mm
*ref*: Reference condition for PSP *in situ* calibration
*t*: Stagnation flow condition
*u*: Upstream of a shock wave
*W*: Total width of the scramjet inlet-isolator model, mm
*x*: *x*-coordinate, mm
*z*: *z*-coordinate, mm
0: Station 0 at freestream
3: Station 3 at isolator exit
*∞*: Free stream
